# Characterizing parasitic nematode faunas in faeces and soil using DNA metabarcoding

**DOI:** 10.1186/s13071-021-04935-8

**Published:** 2021-08-21

**Authors:** Marie Louise Davey, Kjersti Selstad Utaaker, Frode Fossøy

**Affiliations:** 1 Norwegian Institute for Nature Research (NINA), Torgarden, PO Box 5685, 7485 Trondheim, Norway; 2 Faculty of Bioscience and Aquaculture, Nord University, Bodo, Norway

**Keywords:** Gastrointestinal parasitic nematode, Metabarcoding, Amplicon sequencing, Biomonitoring, Parasite, NC1–NC2 primers, Reindeer

## Abstract

**Background:**

Gastrointestinal parasitic nematodes can impact fecundity, development, behaviour, and survival in wild vertebrate populations. Conventional monitoring of gastrointestinal parasitic nematodes in wild populations involves morphological identification of eggs, larvae, and adults from faeces or intestinal samples. Adult worms are typically required for species-level identification, meaning intestinal material from dead animals is needed to characterize the nematode community with high taxonomic resolution. DNA metabarcoding of environmental samples is increasingly used for time- and cost-effective, high-throughput biodiversity monitoring of small-bodied organisms, including parasite communities. Here, we evaluate the potential of DNA metabarcoding of faeces and soil samples for non-invasive monitoring of gastrointestinal parasitic nematode communities in a wild ruminant population.

**Methods:**

Faeces and intestines were collected from a population of wild reindeer, and soil was collected both from areas showing signs of animal congregation, as well as areas with no signs of animal activity. Gastrointestinal parasitic nematode faunas were characterized using traditional morphological methods that involve flotation and sedimentation steps to concentrate nematode biomass, as well as using DNA metabarcoding. DNA metabarcoding was conducted on bulk samples, in addition to samples having undergone sedimentation and flotation treatments.

**Results:**

DNA metabarcoding and morphological approaches were largely congruent, recovering similar nematode faunas from all samples. However, metabarcoding provided higher-resolution taxonomic data than morphological identification in both faeces and soil samples. Although concentration of nematode biomass by sedimentation or flotation prior to DNA metabarcoding reduced non-target amplification and increased the diversity of sequence variants recovered from each sample, the pretreatments did not improve species detection rates in soil and faeces samples.

**Conclusions:**

DNA metabarcoding of bulk faeces samples is a non-invasive, time- and cost-effective method for assessing parasitic nematode populations that provides data with comparable taxonomic resolution to morphological methods that depend on parasitological investigations of dead animals. The successful detection of parasitic gastrointestinal nematodes from soils demonstrates the utility of this approach for mapping distribution and occurrences of the free-living stages of gastrointestinal parasitic nematodes.

**Graphical abstract:**

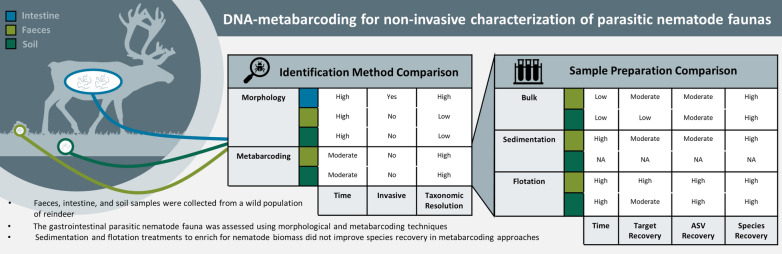

**Supplementary Information:**

The online version contains supplementary material available at 10.1186/s13071-021-04935-8.

## Background

Gastrointestinal parasitic nematodes (GINs) are of global concern for human and animal welfare and health. They are also important in a food production context, as they may cause reduced growth, morbidity, and mortality in livestock and thus generate significant economic losses due to a decrease in product quality and quantity [1, 2]. In addition, GINs are also increasingly recognized to have significant impacts on fecundity, development, behaviour, and survival of wild vertebrate populations [3–8]. Improved understanding of GIN ecology and the interplay between multispecies GIN communities and their hosts is needed, particularly considering that global changes are expected to have major impacts on GINs [9, 10] and that there is mounting evidence for interspecies and density-dependent effects on GINs [11–13]. However, non-invasive methodologies for the diagnosis and monitoring of GINs in wild populations lag behind those for domestic animals [14].

Conventional monitoring of GINs in wild vertebrate populations at the species level typically requires morphological identification of larval and/or adult nematodes observed during post-mortem investigations of the gastrointestinal tract from individuals that have been hunted, culled, lethal sampled, or died of natural causes [5, 15, 16]. Non-invasive conventional methods for monitoring GINs have been limited to faecal egg and larvae counts or coproculture of extracted eggs for species identification [14]. The precision and sensitivity of egg and larvae counts can be substantially increased when the nematode biomass in the samples is concentrated, typically by sedimentation or flotation methods [17–19]. However, all monitoring methods based on morphological identifications are to some degree limited, as small-bodied taxa, including nematodes, are comparatively difficult to monitor using morphological methods. Taxonomic expertise is often rare and of limited availability, species identification is time- and labour-intensive, and there is a paucity of taxonomic characters at some life history stages (eggs and larvae), meaning fewer individuals can be assigned to the species level [20]. A variety of PCR-based molecular methods have been developed for the detection, identification, and quantification of specific GIN species and have the advantage of being able to reliably identify these species at any life stage [21, 22]. These molecular methods have proved useful in diagnostic settings, as well as for investigating the epidemiology and population genetics of these parasites [22]. More recently, high-throughput amplicon sequencing (metabarcoding) targeting different gene regions has been used to investigate the diversity and genetic structure of both free-living and parasitic nematode populations in a variety of environments and hosts (18S rDNA: [23–28]; cytochrome oxidase 1 (CO1): [23, 29]; cytochrome B (cytB): [30]; ITS2 rDNA: [31–34]. Metabarcoding approaches allow the simultaneous identification of a wide range of GIN species, irrespective of life stage, from bulk environmental and faecal samples in a more time- and cost-effective manner than morphological surveys, making it an attractive method for identifying and monitoring occurrence of actual species of GINs in wild vertebrate populations. However, methods for metabarcoding of GINs are still under optimization, and it remains unclear what methodological steps can be used to maximize the precision and sensitivity of metabarcoding assays. For example, concentrating nematode biomass prior to DNA extraction as is done in morphological surveys could minimize the presence of non-target organisms in the sample, increasing the probability of amplifying and sequencing target organisms, which would effectively increase sampling effort and the probability of detecting rare species.

Metabarcoding of the internal transcribed spacer 2 (ITS2) region of rDNA using primers specific to clade V parasitic nematodes has been demonstrated as an effective method for characterizing GIN species in both domestic and wild ruminant populations [33–35]. Here, we evaluate the potential of using metabarcoding for monitoring the presence of parasitic nematode species in wild reindeer both at the individual level, from faecal samples, and at the environmental level from soil samples. We evaluated (1) if metabarcoding provides comparable results to pathological and morphological identification, (2) whether conventional flotation and sedimentation methods for extracting nematode biomass from bulk faeces and soil samples can improve the sensitivity of metabarcoding, and (3) whether gastrointestinal parasitic nematodes can be detected on an environmental level during their free-living stages using soil samples.

## Methods

### Sampling and sample processing

#### Soil samples

Topsoil was collected at suspected hot-spots of ungulate interaction as well as control locations with no obvious signs of animal activity (e.g. defecation, tracks, signs of grazing) observed.

The top layer of soil was scraped carefully away before a spoonful of soil was collected every 20 cm in a fan-like pattern along a 2-m gradient in the runoff direction from the centre of the site, collecting approximately 1 L of soil in total. The samples were frozen at −12 °C before transport and processed after thawing at 4 °C. After thawing, the samples were sifted through a 2-mm sieve to remove larger objects such as stones and twigs, and thoroughly mixed before further processing.

#### Gastrointestinal tracts and faeces

During the 2018 annual hunt of the wild reindeer populations of Knutshø and Forelhogna, Norway, samples of abomasa, proximal duodena, caeca, and faeces were collected from harvested animals in collaboration with hunters and local wildlife officials. The faecal samples were collected directly from the rectum, stored in separate containers, and cooled at 4 °C before processing. The gastrointestinal organs were bagged separately and frozen at −12 °C before thawing and processing.

### Parasitological procedures

A variety of flotation and sedimentation preprocessing steps can be used to extract eggs, larvae, and adult worms from soil and faeces in order to improve the sensitivity and efficiency of monitoring using morphological inventory methods [36, 37]. In this study, the procedures described by Hansen and Perry [37] were followed to identify the GIN communities present in abomasum, duodenum, cecum, faeces, and soil samples.

#### Abomasum

After thawing at room temperature, the abomasal contents and wall were washed with tap water into a bucket, and the volume was adjusted to 2 L. Four 50-ml, 2.5% aliquots were then taken from the middle of the bucket while stirring and placed in 50-ml centrifuge tubes. The tubes were allowed to sediment for 30 min, after which the supernatant was carefully removed. The tube was again topped off with tap water to make the suspension clearer. Each 50-ml subsample was portioned into a rectangular plastic dish with a counting grid and examined under a magnifying lamp, and adult nematodes were picked out and counted. The total abomasal worm burden was estimated based on subsample counts representing 2.5% to 10% of the total abomasal content. Adult worms were stored in petri dishes containing 70% ethanol and examined on an object glass with a drop of physiological saline solution under a cover slip at ×20–100 magnification for both sex and species determination. Species identification was based on the morphological features of the male bursal organs, and spicules, dorsal ray, and gubernaculum features [38, 39]. Nematode infection was estimated per species as described by Bye [40]. Briefly, all adult worms were counted, males were assigned to species or morphotype, and the proportion of male specimens of each species was used to extrapolate the total population of that species. This method assumes no species-specific differences in sex ratios in nematodes. A selection of adult worms were stored in 70% ethanol, some of which were later used for generating reference sequences (see below).

#### Duodenum

The proximal part of the duodenum was cut longitudinally and the contents washed with tap water. The suspension was poured through a sieve with a pore diameter of 116 µm, and the sieve was inspected visually. Nematodes with structures resembling worms of the Nematodirinae family were picked out with fine tweezers and further examined under the microscope at ×20–100 magnification for species identification, and subsequently stored in 70% ethanol. Only presence data was recorded, as different lengths of the duodenum were sampled from each individual, making abundance data non-comparable. Species determinations were made as described above using Fruetel et al. [41] and Hoberg et al. [42] as taxonomic references.

#### Cecum

The cecum was opened with scissors, and the contents were spread out. As nematodes in the large intestines are visible to the naked eye, they were picked up with forceps and placed in a petri dish containing 70% ethanol, counted, and then further examined with a magnifying lamp and microscope at ×40. Species determinations were made as described above using Taylor et al. [43] as a taxonomic reference.

#### Faeces

Endoparasitic egg and oocyst occurrence and abundance were estimated using a modified McMaster technique [36]. McMaster counting chambers (Whitlock Universal, Australia) were filled with 2.5 ml of the faeces and saline solution suspension, and the whole slide was read at ×40 and ×100 magnification for detection and quantification of parasite eggs and oocysts. Some eggs and oocysts may be identified to genus level (*Moniezia* sp., *Trichuris* sp., *Nematodirus* sp., and *Eimeria* sp.) based on morphological characteristics, though a number of gastrointestinal nematode eggs can only be identified to order, given morphological similarities and size overlap. Therefore, these eggs were grouped and characterized as strongylid eggs.

The Baermann technique was used to isolate, quantify, and identify L1 stage larvae in the faeces [36]. A 10-g faecal sample, wrapped in gauze, was suspended for a minimum of 12 h in tepid water at room temperature. The bottom 10 ml of sediment was aspirated and centrifuged (at 1500×*g* for 3 min). The supernatant was then aspirated to the 1-ml mark, and a 100-μl subsample of the sediment examined at ×100 magnification for larvae. The larvae were recorded as hatched GIN larvae, and L1 stages of the lungworm (*Dictyocaulus* spp.) and brainworm (*Elaphostrongylus rangiferi*). The number of larvae per gram faeces (LPG) was estimated from the subsample count.

#### Soil

For isolation of parasite eggs and larvae, sifted soil was prepared according to Steinbaum et al. [44] with slight modifications: 15 g of soil was transferred to a 50-ml centrifugation tube and filled with 35 ml 1% 7× detergent. The tube was shaken by hand for 2 min before further filling it with detergent to 45 ml. After standing overnight, the tube was shaken for 1 min, and the contents sifted through a 500-µm sieve into a beaker. The sieve and tube were washed with detergent into the beaker to a volume of 150 ml, and the contents after sedimentation for 1 h were divided into two aliquots in 50-ml centrifugation tubes and filled with detergent to 40 ml before centrifuging for 10 min at 1000 rcf and then discarding the supernatant. Five millilitres of NaCl/ZnCl_2_ solution (specific gravity 1.3 g) was added to the remaining pellet, and the centrifuge tube was vortexed for 30 s and topped with the salt solution up to 40 ml. The tubes were centrifuged for 5 min at 1000 rcf, and the supernatant was sieved through a 10-µm mesh. The mesh was thoroughly washed with detergent and distilled water, and the suspension from the washing step was transferred to a 50-ml centrifuge tube. This step was repeated twice. The tubes were then centrifuged at 1000 rcf for 10 min, and the pellets were combined until one centrifuge tube per soil sample remained. The supernatant was carefully discarded until a 1-ml pellet remained, which was pipetted on a Sedgwick rafter slide and read at ×40 and ×100 magnification for detection and quantification of parasite eggs.

### DNA metabarcoding sample preparation

#### Faeces

DNA metabarcoding was conducted on 3-g faecal subsamples that were processed in three ways prior to DNA isolation:

1. Untreated faeces: the 3-g subsample was directly transferred to a 50-ml FastDNA™ Spin Kit for Soil (MP Biomedicals™) tube.
2. Concentrated faeces: three 3-g subsamples were homogenized in 57 ml water and sieved through a ≈ 1000-µm sieve. The suspension was then divided into three 15-ml tubes and centrifuged at 1550 rcf for 3 min, after which the supernatants were discarded. Finally, 300 µg of the remaining pellet was transferred from one of the centrifuge tubes to a 2-ml FastDNA™ Spin Kit for Soil (MP Biomedicals™) tube.
3. Faecal flotation: the concentrated pellet from one of the centrifuge tubes prepared above in (2) was resuspended in NaCl/ZnCl_2_ solution (specific gravity 1.3 g), then vortexed until the pellet was dissolved into a suspension. The suspension was centrifuged again at 1550 rcf, and the supernatant was sieved through filter fabric (SEFAR^®^ MEDIFAB) with a pore diameter of 10 µm. The filter was washed with a 1% ES 7X™ cleaning solution (MP Biomedicals™) diluted with distilled water, and the wash water was transferred to 50-ml centrifuge tubes. After centrifugation for 10 min at 1550 rcf, the supernatant was carefully discarded and the pellet transferred into a 2-ml FastDNA™ Spin Kit for Soil tube.

#### Soil

DNA metabarcoding was conducted on soil samples that were prepared in two ways:

1. Sieved soil: approximately 15 ml of soil sieved at 2 mm was directly transferred into a 50-ml FastDNA™ Spin Kit for Soil tube.
2. Concentration by flotation: the 1-ml suspended pellet that was used for enumeration of eggs and larvae (see above “Parasitological procedures”: Soil) was transferred to a 2-ml FastDNA™ Spin Kit for Soil tube.

### DNA extraction and sequencing

DNA was extracted from 3 g bulk faeces and 15 ml soil samples using the FastDNA™ Spin Kit for Soil (MP Biomedicals, Germany) in 50-ml volumes according to the manufacturer’s specifications (Protocol Revision: #116560200–201908/#116560000–201908). Faeces and soil samples that had undergone flotation or sedimentation treatments were extracted using the FastDNA™ Spin Kit for Soil (MP Biomedicals, Germany) in 2-ml volumes according to the manufacturer’s specifications (Protocol Revision: #116560200–201908/#116560000–201908). The ITS2 region of ribosomal DNA (rDNA) was amplified using the NC1 and NC2 primer set which targets the clade V group of parasitic nematodes [49]. PCR reactions were conducted in 25-µl volumes containing 1× KAPA HiFi HotStart ReadyMix (Roche, Switzerland), 0.2 µM of the forward and reverse primers, and 25 ng template DNA. PCR conditions consisted of an initial denaturing step of 5 min at 95 °C, followed by 35 cycles of 1 min at 95 °C, 1 min at 54 °C, and 1 min at 72 °C with a final elongation step of 5 min at 72 °C. Amplicon concentrations were normalized using a SequalPrep™ normalization plate (Thermo Fisher Scientific, Germany) and used as template for indexing using the Nextera XT Index Kit (Illumina, USA). Indexing reactions were conducted in 50-µl volumes containing 1× KAPA HiFi HotStart ReadyMix (Roche), 1 µM of the forward and reverse indexing primers, and 5 µl of normalized template DNA. PCR conditions for indexing consisted of an initial denaturing step of 3 min at 95 °C followed by eight cycles of 30 s at 95 °C, 30 s at 55 °C, and 30 s at 72 °C, and a final elongation step of 5 min at 72 °C. The indexed samples were also normalized using a SequalPrep™ normalization plate, pooled in equal volumes, and sequenced in a paired-end 300-bp run on the Illumina MiSeq sequencing platform at the Genomics Core Facility (GCF), Norwegian University of Science and Technology (NTNU), Trondheim, Norway.

### Bioinformatics and statistical analyses

Sample demultiplexing and adapter removal was performed using the MiSeq Reporter on the Illumina MiSeq sequencing platform. Primer sequences were identified and removed from both the 5′ and 3′ ends of forward and reverse reads using cutadapt v.1.9.1 [45], allowing up to 15% mismatch across the length of the primer. Quality filtering, error correction, and chimera detection were all conducted using the DADA2 v.1.12 package for R [46]. Reads were quality-filtered to remove all sequences with ambiguous bases, > 2 expected errors in the forward direction, > 4 expected errors in the reverse direction, and length < 50 bp after truncation at the first instance of a base with a quality score < 20. Error rates were estimated for forward and reverse sequences, forward and reverse reads were merged with a minimum overlap of 30 bp, and amplicon sequence variants (ASVs) were inferred for each sample. Chimeric sequence variants were assessed on a per-sample basis, as chimeric events occur at the individual PCR level. If a sequence variant was flagged as chimeric in more than 90% of the samples in which it occurred, it was removed. To assess the possibility of taxa not being detected due to the variable length of the nematode ITS2 region, the forward reads were also analysed in parallel as described above, omitting the merging step. Taxonomy was assigned to ASVs using the naïve Bayesian classifier [47] implemented in DADA2 and a custom version of the Nematode ITS2 v.1.0.0 database [34, 48] (http://www.nemabiome.ca) including an additional 19 reference sequences from adult nematodes and eggs identified during morphological examinations of the samples in this study (GenBank accession no. MZ478650-MZ478668). DNA was extracted from dried reference specimens after homogenization using the DNeasy Blood & Tissue Kit (Qiagen, Germany), and the ITS2 rDNA was amplified using the primers NC1 & NC2 [49]. Excess primers and dNTPs were removed from the amplicons using ExoSap-IT™ (Applied Biosystems, USA), which were further sequenced from both ends using the BigDye Terminator v1.1 kit (Applied Biosystems, USA) on a 3500xl Genetic Analyzer (Applied Biosystems, USA). A minimum confidence estimate of 80% was required for successful assignment against the custom database at any given taxonomic level. Each ASV was also subjected to a BLAST search against the NCBI nucleotide non-redundant database. Any ASV with a best BLAST match to a lineage outside the phylum Nematoda or that could not be assigned with confidence > 80% at the phylum level was designated a non-target amplification and excluded from further analyses. Those ASVs not successfully assigned at the species level were further clustered into operational taxonomic units (OTUs) at 97% identity using the vsearch algorithm [50]. Each OTU was given the taxonomic assignment of the most abundant ASV in the OTU.

All statistical analyses were conducted in the R statistical environment [51] on the data set derived from the merged forward and reverse reads. ANOVA was used to assess differences in the proportion of target reads obtained in soil vs faecal samples. General linear models were used to assess differences in ASV and species recovery between treatment methods for both faeces and soil samples, with the log-transformed sequencing depth included as a fixed effect and biological sample included as a random effect in both cases.

## Results

### GIN parasites in faeces and gastrointestinal tracts

We detected parasitic species of the genera *Cooperia*, *Elaphostrongylus*, *Haemonchus*, *Nematodirus*, *Ostertagia*, *Spiculopteragia*, *Teladorsagia*, *Trichostrongylus*, and *Trichuris* in reindeer faecal and intestinal samples using morphological and/or metabarcoding methods. All egg, larvae, and oocyst counts from the McMaster and Baermann analyses of faeces were low (Table 1), which may indicate a low production rate of eggs or larvae due to time of sampling (season) or a small number of egg- or larvae-producing adult females in the gastrointestinal system and lungs.

**Table 1:** Results from faecal analysis by the McMaster and Baermann technique

Parasite group	Individual 1	Individual 2	Individual 3		Individual 4	Individual 5
Strongylid EPG	20	70	180	220		30
Nematodirinae EPG	–	–	–	10		–
*Nematodirus battus* EPG	–	–	–	–		50
*Trichuris* sp. EPG	–	10	–	–		–
*Eimeria* sp. OPG	–	–	–	–		400
*Dictyocaulus eckerti* LPG	–	10	1.8	–		–

*EPG* eggs per gram, *OPG* oocysts per gram, *LPG* larvae per gram faeces

–: not detected

By contrast, the total adult abomasal trichostrongylid worm burden was in the range of low to moderate (Table 2). As the sampled proximal duodena had different lengths, and in one case had been nearly completely scavenged by ravens prior to sampling, parasites were identified to species but not quantified. Faecal egg counts and total abomasal worm burden were not significantly correlated (*p* > 0.05, Additional file 2: Figure S1a).

**Table 2:** Results from analysis of adult gastrointestinal parasites in abomasa, duodenum, and ceca

	Individual 1	Individual 2	Individual 3	Individual 4	Individual 5
Abomasal parasites					
*T. circumcincta*	90	350	–	190	410
*O. gruehneri*	770	–	4320	3020	–
*O. arctica*	–	–	90	470	–
Total abomasal adult worm burden	860	350	4410	3680	410
Duodenal parasites					
*N. tarandi*	–	Positive	–	na	–
*N. longissimespiculata*	–	Positive	–	na	–
*N. battus*	–	–	–	na	Positive
Cecal parasites	–	–	–	–	–

–: not detected; positive: parasite found, but not possible to quantify; na: duodenum missing from sample

**Fig. 1:**
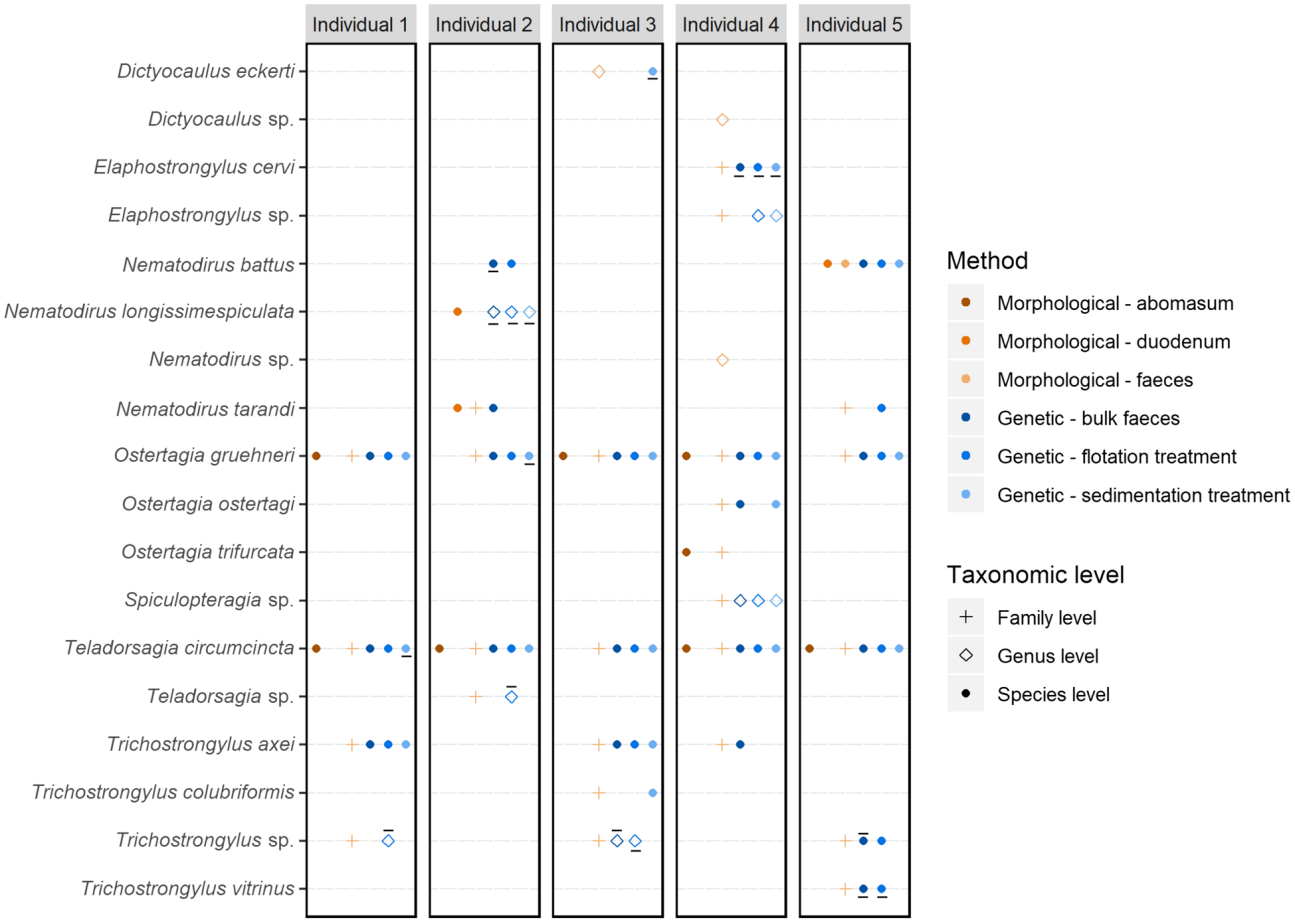
Comparison of five reindeer individuals’ parasitic nematode communities detected using morphological and molecular methods. Morphological surveys included pathological examinations of the abomasum and duodenum in addition to flotation of eggs from faeces. Molecular surveys used amplicon sequencing of faeces samples either directly or after enrichment treatments by flotation or sedimentation. Point type indicates the lowest taxonomic level a method successfully identified the taxon at. A solid line under the point indicates it was recovered only from the forward reads. A solid line over the point indicates it was recovered only from the combined forward and reverse reads

The nematode communities identified from faecal samples using molecular and morphological methods were largely congruent (Fig. 1). Those species identified from parasitological investigations of the abomasum, duodenum, and faeces were also recovered with the metabarcoding approach. However, it must be noted that *Dictyocaulus eckerti*, *Elaphostrongylus cervi*, and *Trichostrongylus vitrinus* were only recovered when the forward reads were analysed without merging with the reverse reads from the fragments (Fig. 1), which allowed for the retention of more read pairs than in the merged data set (Additional file 1: Table S1). In particular, molecular methods allowed species-level identification of a greater diversity of nematodes, particularly of members of the suborder Strongylida from faecal samples. However, the proportional abundance of a given species in the GIN community based on parasitological investigations of the abomasum and duodenum was not significantly correlated with the proportional abundance of DNA sequences of that species determined from metabarcoding of faecal samples from the same individual (Additional file 3: Figure S2b).

### GIN parasites in soils

Species of the genera *Nematodirus*, *Ostertagia*, and *Teladorsagia* were detected in soil samples using the metabarcoding approach, while traditional parasitological investigations identified eggs of *Nematodirus* and *Moniezia* (Table 3). Morphological identification confirmed the presence of *Nematodirus battus* in four of six soil samples analysed, and sequencing of both bulk and flotation-treated soils detected the species in the same samples (Additional file 4: Figure S3). However, there was no significant correlation between the number of *N. battus* eggs identified morphologically after flotation treatment of the soil and the proportional abundance of *N. battus* reads in the metabarcoded soil samples (Additional file 4: Figure S3).

**Table 3:** Results from enumeration of eggs and strongylid larvae in soil samples

	Sample 1	Sample 2	Sample 3	Sample 4	Sample 5	Sample 6
*N. battus* eggs	15	10	4	0	–	–
Nematodirinae egg	–	–	–	1	–	–
Larvae	140	4	25	1	69	7
*Moniezia* sp. eggs	–	–	–	1	–	–

### Sample handling for metabarcoding

**Fig. 2:**
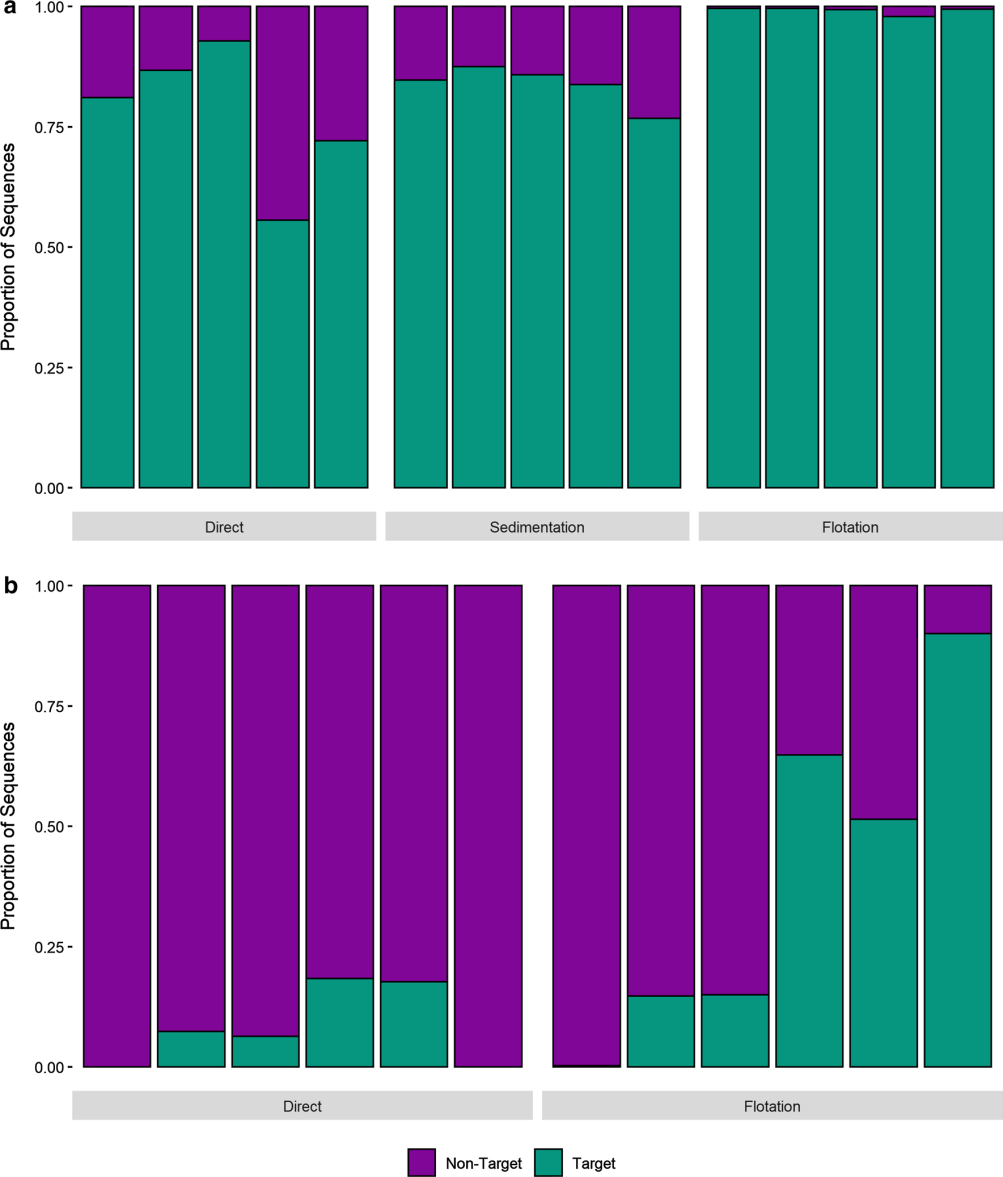
Target sequence recovery from faeces and soil samples. Recovery of parasitic nematode reads (target) from faeces (**a**) and soil (**b**) samples where DNA has been isolated either directly or after flotation or sedimentation enrichment treatments. Each bar represents an individual faeces or soil sample. Samples are presented in the same order for each method

**Fig. 3:**
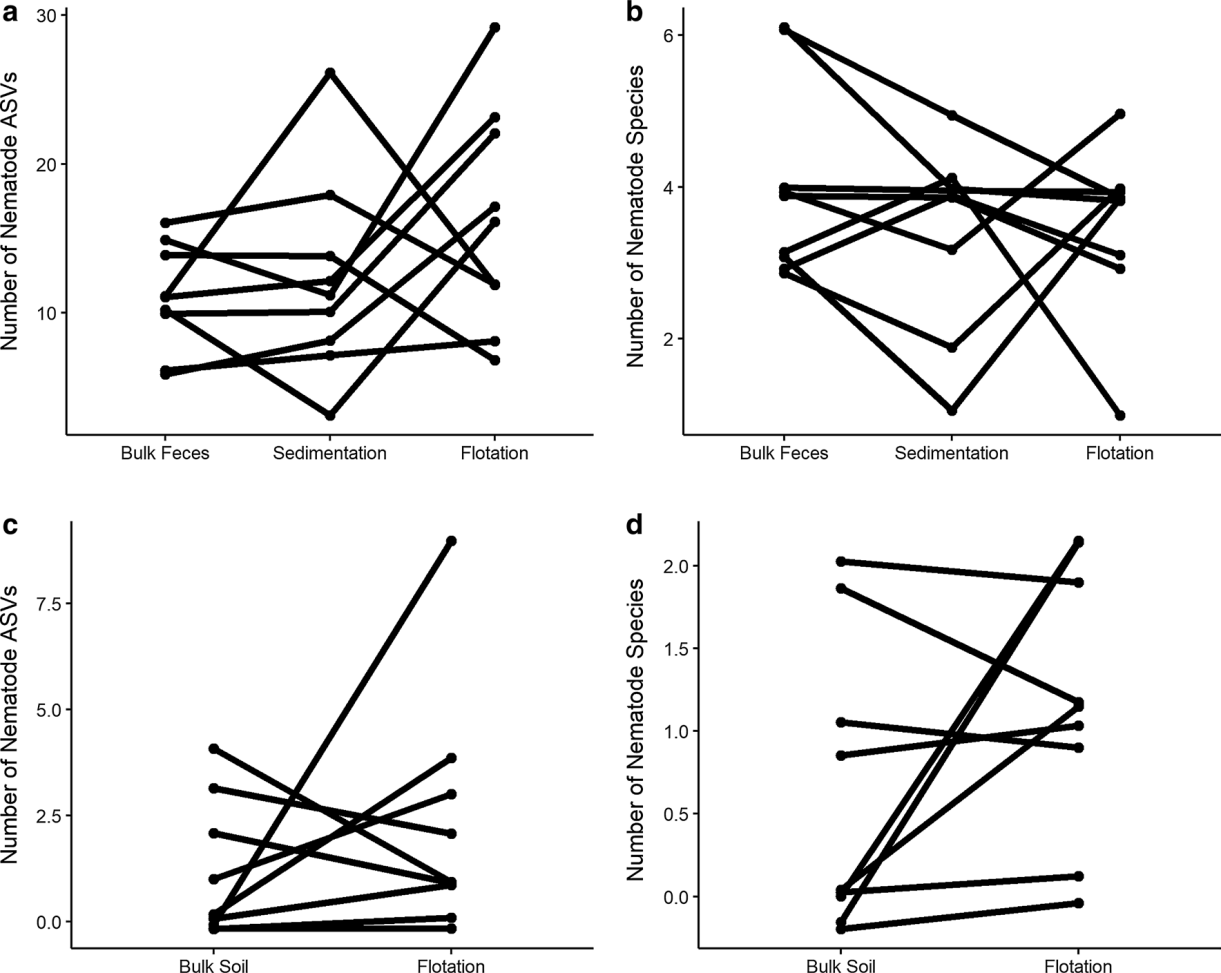
Comparison of species and ASV recovery using different sample preparation methods. Recovery of parasitic nematode ASVs (**a**, **c**) and species (**b**, **d**) from faeces (**a**, **b**) and soil (**c**, **d**) samples after amplicon sequencing of DNA isolated directly or after enrichment treatment by flotation or sedimentation. Each line connects the points representing the treatment combinations for a specific biological sample

A total of 11,084,764 sequences were generated across samples in the MiSeq run. Of those sequences that passed quality filtering, 79.7% could be positively identified as belonging to the phylum Nematoda, and the remaining 21.7% originated from non-target amplification. In none of the treatments was the proportion of target nematode sequences correlated with the total number of GIN larvae or eggs detected using traditional parasitological methods (*p* < 0.05, Additional file 2: Figure S1b, c). The proportion of clade V Nematoda reads recovered was significantly higher in faeces samples than in soil samples (89.8 ± 11.0% and 19.6 ± 28.4%, respectively, *p* < 0.001, Fig. 2). Flotation and sedimentation treatments of faecal samples prior to DNA isolation significantly increased the proportion of target Nematoda reads recovered (*p* = 0.003, Fig. 2a) and decreased the proportion of non-target ASVs recovered compared to bulk samples. However, neither treatment significantly increased the number of nematode ASVs (*p* = 0.747) or species (*p* = 0.811) recovered per sample (Fig. 3a, b). ASV recovery (*p* < 0.001) correlated with sequencing depth, although species recovery did not (*p* = 0.101, Additional file 4: Figure S3). Flotation treatment also significantly increased the proportion of target Nematoda reads recovered from soil samples (*p* = 0.035, Fig. 2b) and decreased the proportion of non-target ASVs sequenced. However, the number of target clade V Nematoda ASVs (*p* = 0.820) and species (*p* = 0.323) recovered from soil samples did not vary significantly between the methods (Fig. 3c, d). ASV recovery (*p* < 0.001) correlated with sequencing depth, although species recovery did not (*p* = 0.290, Additional file 5: Figure S4).

The GIN communities recovered by metabarcoding were largely consistent between DNA isolated from bulk faeces samples compared to faeces subjected to flotation or sedimentation processing prior to DNA isolation (Fig. 1), and there were no consistent patterns of preferential enrichment for specific taxa by either (Additional file 3: Figure S2a). Similarly, sequencing of both bulk and flotation-treated soils detected *Nematodirus battus* in the same soil samples as flotation tests and morphological identification (Additional file 4: Figure S3).

## Discussion

### Metabarcoding for assessing GIN communities in faeces

Molecular methods, including metabarcoding, have been identified as promising tools for non-invasive monitoring of GINs in wild vertebrate populations [33, 35, 52, 53]. We demonstrate here that metabarcoding provides a viable alternative to traditional parasitological investigations for detecting GIN species in wild populations of reindeer. The nematode communities identified from faecal samples using molecular and morphological methods were largely congruent: species identified from parasitological investigations were also recovered with the metabarcoding approach. This has been observed for other species, including horses and gorillas [32, 54, 55]. However, it must be noted that false negatives were detected for three species in the data set where forward and reverse reads were merged. In the case of *Dictyocaulus eckerti* and *Elaphostrongylus cervi*, the ITS2 region is  > 480 bp and is likely too large to allow successful forward and reverse read merging with the Illumina paired-end 300-bp chemistry, resulting in false-negative results in the merged data. *Trichostrongylus axei* has a more moderately sized ITS2 fragment (< 300 bp) and was more likely excluded from the merged data due to, for example, low-quality reverse reads eliminating all *T. axei* read pairs. This is supported by the retention of more reads post-quality filtering from the forward reads alone than from the merged read pairs. The identification of false negatives in the data that can be directly attributed to bioinformatic decisions highlights the need for consideration of a priori knowledge of the target organisms in order to optimize the metabarcoding methodology for these types of assays.

Compared to conventional methods using morphological identification of adult worms, larvae, and eggs, molecular methods using faeces have higher throughput, are repeatable, provide species-level identifications of all GIN life stages, and have no dependence on lethal sampling [14, 31, 54]. Although a linear scaling between metabarcoding sequence abundance and larval abundance in faecal samples has been documented in several cases [31, 55], we observe no correlation between egg counts and the proportion of nematode reads recovered, although it must be noted that our observations are based on only five individuals. The reliability of metabarcoding as a quantitative estimate of parasitic GIN load is complicated by the fact that GINs have complex life cycles, where many species often undergo hypobiosis as an obligate or alternative part of their life cycles, and thus egg production and subsequent detection in faeces by metabarcoding may be dependent on the stress and/or immunity status of the animal. This is clearly reflected in that the total adult worm burden in the abomasa in this study varied between individuals by an order of magnitude, but was correlated with neither the faecal egg count, nor the relative abundance of target nematode sequences obtained by metabarcoding the faeces. Although the proportional abundances of species recovered from faecal samples have previously been found to correlate with the proportional abundance of larvae in the faeces [32, 55], we find no correlation between the proportional abundances of species in faecal samples and the proportional abundance of adult worms of that species in the abomasum. In particular, the quantitative differences between counts of adult individuals from the gastrointestinal system and the inferred abundance from faecal samples may be exacerbated by species-specific variation in life histories or by the fact that faecal samples were collected in early autumn, a season where GIN egg counts are not typically high. This suggests that while assessing GIN communities from faecal samples has the advantage of being non-invasive and non-lethal, it does not necessarily provide good estimates of actual parasitic GIN loads. However, our observations are based on very few individuals (5), and a much more comprehensive and intensive study combining morphological quantification of adult larvae from gastrointestinal samples with both parasitological investigations and metabarcoding of faecal samples is required to fully explore whether metabarcoding sequence abundance can reflect actual parasitic GIN loads.

While molecular methods can allow species level identification of life stages with relatively few morphological characters, they do not indicate whether the parasite is living, viable, and capable of infecting the next host. As such, metabarcoding can be used for confirming the presence of a specific GIN species, mapping the distribution of GIN species, and monitoring nemabiome species richness in wild hosts. Our results suggest metabarcoding cannot provide effective estimates of parasite burden and the total parasite impact on their host, especially when other pathogenic and production-limiting parasites such as protozoans, cestodes, and trematodes are not included. However, more detailed investigations including larger sample sizes, technical replicates, and testing for false-positive/negative detections are required to properly assess the potential of metabarcoding for quantitative assessments of GIN populations.

To our knowledge, this study represents the first report of a naturally acquired *Haemonchus* sp. infection in wild reindeer. Although the adult worm was not found in the abomasum, the parasite may have been present as an egg identified as belonging to the order Strongyloidea. *Nematodirus battus* in wild reindeer was recently reported for the first time by [56], and is also confirmed here with both morphological and metabarcoding data, demonstrating the utility of metabarcoding for surveying and monitoring GIN parasite diversity in wild populations.

### Metabarcoding for assessing GIN communities in soils

Metabarcoding of DNA extracted from soil samples in grazing ranges of wild reindeer populations successfully detected the primary components of the local reindeer GIN fauna (*Teladorsagia circumcincta*, *Ostertagia gruehneri*, *Nematodirus battus*). Comparisons with morphological identifications from the same soil samples suggests that the molecular method has a comparable degree of sensitivity to morphological identification of extracted eggs, with the added benefit of better taxonomic resolution for more groups, such as members of the order Strongylida, though with the disadvantage of not producing quantitative data or information on viability, which are crucial factors when assessing parasite impact on its host. Nonetheless, successful detection of these parasites from soil samples in the grazing areas of a wild ruminant highlights the utility of this approach for mapping the spatial distribution of the parasites during their free-living stages, allowing for improved understanding of transmission routes, infection pressures, and GIN population dynamics. The elucidation of potential presence of certain GIN transmission stages in areas where wild ruminants may be stationary for prolonged periods (e.g. calving grounds, confined feeding grounds, mineral licks) could hopefully contribute to subsequently improved surveillance and management of wild ruminant population health.

### Sample handling for metabarcoding of GIN communities

Conventional morphological identification-based methods for characterizing GIN communities typically require pretreatment of biological samples to extract eggs, larvae, and adult worms and concentrate GIN biomass for subsequent quantification and identification. These identifications are commonly performed on faecal samples where species identification to species and even genus level is difficult, and may require both as-fresh-as-possible material as well as time-consuming cultivation steps to ensure the accurateness of the results. Although these pretreatments can be both labour intensive and costly, they significantly increase the sensitivity of nematode community surveys both from faeces and soil substrates [17–19]. We evaluated the potential for conventional concentration treatments to increase effective sampling effort (i.e. the recovery of target Nematoda sequences), thereby increasing the sensitivity of amplicon sequencing-based detection of parasitic clade V nematodes from both faeces and soil samples as compared to direct sequencing of the biological material. Although enrichment for nematode biomass through flotation or sedimentation treatments prior to DNA isolation significantly increased the number of target clade V Nematoda reads recovered from both faeces and soils, the number of species and genetic variants detected were not significantly increased by these treatments. The lack of improved sensitivity after enrichment treatments likely reflects the fact that GIN communities are relatively species-poor, which is a common feature in GIN communities in wild ruminants at these latitudes [57]. In addition, the low diversity observed may be as a result of collecting faecal samples during a season where high egg and larvae output is not expected. The increased non-target amplification observed in the absence of pretreatments to concentrate nematode biomass does not appear to reduce the effective sequencing depth to levels impacting detection success rates at the species or ASV levels. Although pretreatment to enrich nematode biomass in samples may nonetheless increase sensitivity in assays of more diverse nemabiomes than those studied here, continuing advances in sequencing technologies are increasing sequencing yields without concurrent cost increases and will likely provide sufficient sequencing depths without pretreatment, irrespective of GIN community diversity. Therefore, an NC1–NC2 amplicon sequencing-based approach for monitoring parasitic nematode community species richness in animals provides a further cost- and labour-saving advantage over morphology-based approaches by circumventing the need for pretreatment of samples to extract eggs, larvae, and adult worms, thereby simplifying nematode diversity assessment.

## Conclusions

DNA metabarcoding and morphological identification of GIN communities from soil and faeces samples were largely congruent. Although DNA metabarcoding does not provide information on parasite load, viability, and life stage, it has the advantage of providing data with high taxonomic resolution, allowing for non-invasive monitoring of wild populations. Furthermore, DNA metabarcoding can represent a time- and cost-effective method for high-throughput monitoring of GIN communities compared to morphological approaches, as pre-processing samples by flotation or sedimentation to concentrate nematode biomass does not significantly improve species detection.

## Supplementary Information

**Additional file 1: Table S1.** Metabarcoding sequences retained after filtering, denoising, merging and chimera checking. Results are presented for processing of only the forward reads (Forward) as well as processing of the paired end forward and reverse reads together (Merged).

**Additional file 2: Figure S1.** Relationships between total abomasal worm count and **a** faecal egg count and **b** proportion of target nematode metabarcoding sequences, as well as **c** the relationship between faecal egg count and the proportion of target nematode metabarcoding sequences.

**Additional file 3: Figure S2.** Proportional abundances of species recovered from faeces samples after flotation and sedimentation treatments (**a**). Comparison of the proportional abundances of species in GIN communities as determined by morphological identification from abomasum and duodenum samples, and molecular identification from faecal samples (**b**) B: bulk samples, untreated, F: flotation, S: sedimentation.

**Additional file 4: Figure S3.** Detection of *Nematodirus battus* from soil samples using morphology- and molecular-based approaches. Points are sized relative to the number of *N. battus* eggs detected (morphology) or the proportional abundance of *N. battus* reads in the sample (genetic methods).

**Additional file 5: Figure S4.** The relationship between sequencing depth and the number of parasitic nematode ASVs (**a**) and species (**b**) recovered from faeces and soil samples. Statistically significant relationships are indicated with fitted lines.

## Data Availability

The sequence data generated and/or analysed during the current study are available in the NCBI Short Read Archive under project accession number PRJNA742529 (SUB9929863). The morphological data generated and analysed during this study are included in this published article.

## Abbreviations

ASV: Amplicon sequence variant
DNA: Deoxyribonucleic acid
GIN: Gastrointestinal nematode
ITS2: Internal transcribed spacer 2
OTU: Operational taxonomic unit
rDNA: Ribosomal deoxyribonucleic acid
