# Supplementary material for: Characterizing parasitic nematode faunas in faeces and soil using DNA metabarcoding
Source: Parasit Vectors. 2021 Aug 21;14:422. doi: 10.1186/s13071-021-04935-8 (PMC8380370; doi:10.1186/s13071-021-04935-8)
Supplement: Supplementary file 1 — Additional file 1: Table S1. Metabarcoding sequences retained after filtering, denoising, merging and chimera checking. Results are presented for processing of only the forward reads (Forward) as well as processing of the paired end forward and reverse reads together (Merged). [file 13071_2021_4935_MOESM1_ESM.pdf]

Table S1. Metabarcoding sequences retained after filtering, denoising, merging and chimera checking. Results are presented for processing of only the forward reads (Forward) as well as processing of the paired end forward and reverse reads together (Merged).

			Filtering		Denoising R1		Denoising R2		Merged		Nonchimeric		Percent Retained	
**sample**	**sample_type**	**raw**	Forward	Merged	Forward	Merged	Forward	Merged	Forward	Merged	Forward	Merged	Forward	Merged
**sample_1**	bulk_soil	220516	37082	21026	30700	17526		20051		9875	30700	9875	13.9	4.5
**sample_2**	bulk_soil	141456	22703	14439	17273	10657		10190		5404	17256	5404	12.2	3.8
**sample_3**	bulk_soil	30704	4557	2741	2469	1285		1245		270	2469	270	8.0	0.9
**sample_4**	bulk_soil	110560	17583	10393	11798	6946		7724		2545	11782	2545	10.7	2.3
**sample_5**	bulk_soil	330736	53572	32853	44800	27262		26449		18215	43746	17576	13.2	5.3
**sample_6**	bulk_soil	207692	32835	20095	24266	14318		18050		5969	24203	5966	11.7	2.9
**sample_7**	bulk_soil	111364	16526	9338	11155	6634		6354		2228	11151	2228	10.0	2.0
**sample_8**	bulk_soil	141844	22045	13707	15885	9644		9383		3760	15885	3760	11.2	2.7
**sample_9**	bulk_soil	98112	15496	9389	11478	6749		6083		3505	11425	3488	11.6	3.6
**sample_10**	soil_flotation	115732	20918	14253	15883	11805		13098		7736	15883	7736	13.7	6.7
**sample_11**	soil_flotation	234136	41596	30097	33101	25872		28275		12969	31791	12593	13.6	5.4
**sample_12**	soil_flotation	557508	103241	70685	66533	53707		67984		41510	63964	38166	11.5	6.8
**sample_13**	soil_flotation	282180	51199	36067	43155	32521		33752		25043	41495	24449	14.7	8.7
**sample_14**	soil_flotation	94668	16672	11605	10901	8530		9669		7149	10542	6867	11.1	7.3
**sample_15**	soil_flotation	395180	74709	52822	66017	48909		50539		40430	63908	39575	16.2	10.0
**sample_16**	soil_flotation	127888	20370	13196	16785	11088		10955		7656	16709	7656	13.1	6.0
**sample_17**	soil_flotation	134244	22874	13876	18512	11230		11497		7247	18431	7240	13.7	5.4
**sample_18**	soil_flotation	68456	12783	9004	8684	7020		8306		6420	8684	6420	12.7	9.4
**sample_23**	faeces_sedimentation	418008	79785	56735	47109	35948		48701		30384	46235	30172	11.1	7.2
**sample_24**	faeces_sedimentation	342428	62433	44009	39139	31273		40888		25396	38673	25396	11.3	7.4
**sample_25**	faeces_sedimentation	20616	3659	2682	3027	2322		2509		1800	3027	1800	14.7	8.7
**sample_26**	faeces_sedimentation	226780	43328	33452	26692	24058		28373		19841	26098	19635	11.5	8.7
**sample_27**	faeces_sedimentation	159320	28832	21693	19153	16164		19020		12397	18987	12380	11.9	7.8
**sample_32**	faeces_flotation	1194096	226773	179828	130139	120113		158401		94464	123722	92002	10.4	7.7
**sample_33**	faeces_flotation	970184	193200	149467	109673	98654		115238		69203	102113	66786	10.5	6.9

**sample_34**	faeces_flotation	1790856	354187	282270	189107	175299		237464		137657	180502	134037	10.1	7.5
**sample_35**	faeces_flotation	1035328	204589	164549	108798	104273		117434		75686	103029	74074	10.0	7.2
**sample_36**	faeces_flotation	600980	117407	88409	63160	56994		69001		43195	60235	42307	10.0	7.0
**sample_41**	bulk_faeces	102568	18191	12608	13490	10216		11305		9110	13432	9110	13.1	8.9
**sample_42**	bulk_faeces	170068	31017	22966	20255	16949		18903		13030	20079	13030	11.8	7.7
**sample_43**	bulk_faeces	240544	43752	32317	29735	24053		30824		20158	29528	20141	12.3	8.4
**sample_44**	bulk_faeces	201028	34091	22620	23760	16840		20269		14048	23270	13912	11.6	6.9
**sample_45**	bulk_faeces	208984	35706	24791	24146	17960		19896		12734	23888	12606	11.4	6.0
