# Supplementary figures and images for: Characterizing parasitic nematode faunas in faeces and soil using DNA metabarcoding
Source: Parasit Vectors. 2021 Aug 21;14:422. doi: 10.1186/s13071-021-04935-8 (PMC8380370; doi:10.1186/s13071-021-04935-8)

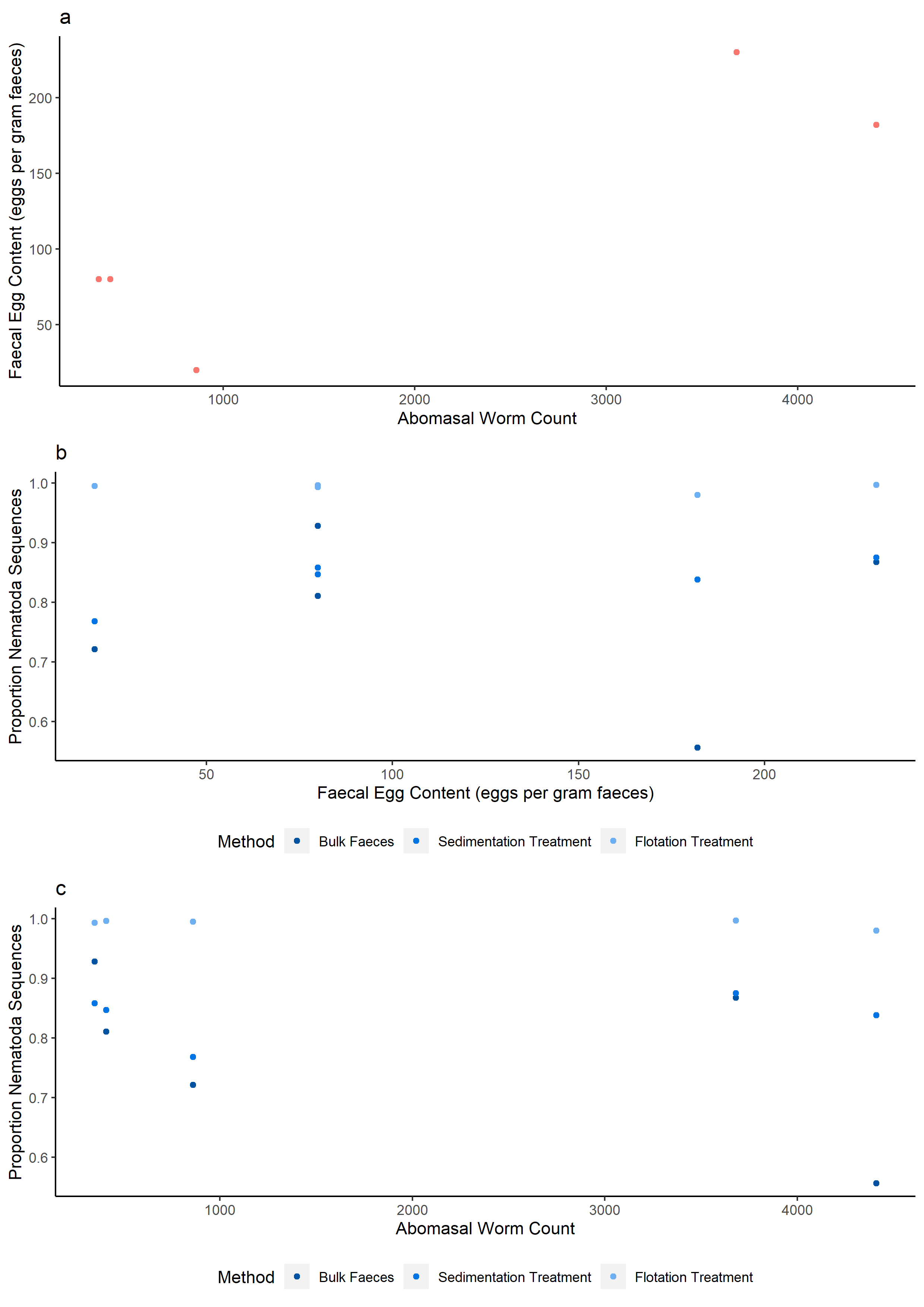

Supplement: Supplementary file 2 — Additional file 2: Figure S1. Relationships between total abomasal worm count and a faecal egg count and b proportion of target nematode metabarcoding sequences, as well as c the relationship between faecal egg count and the proportion of target nematode metabarcoding sequences. [file 13071_2021_4935_MOESM2_ESM.png]

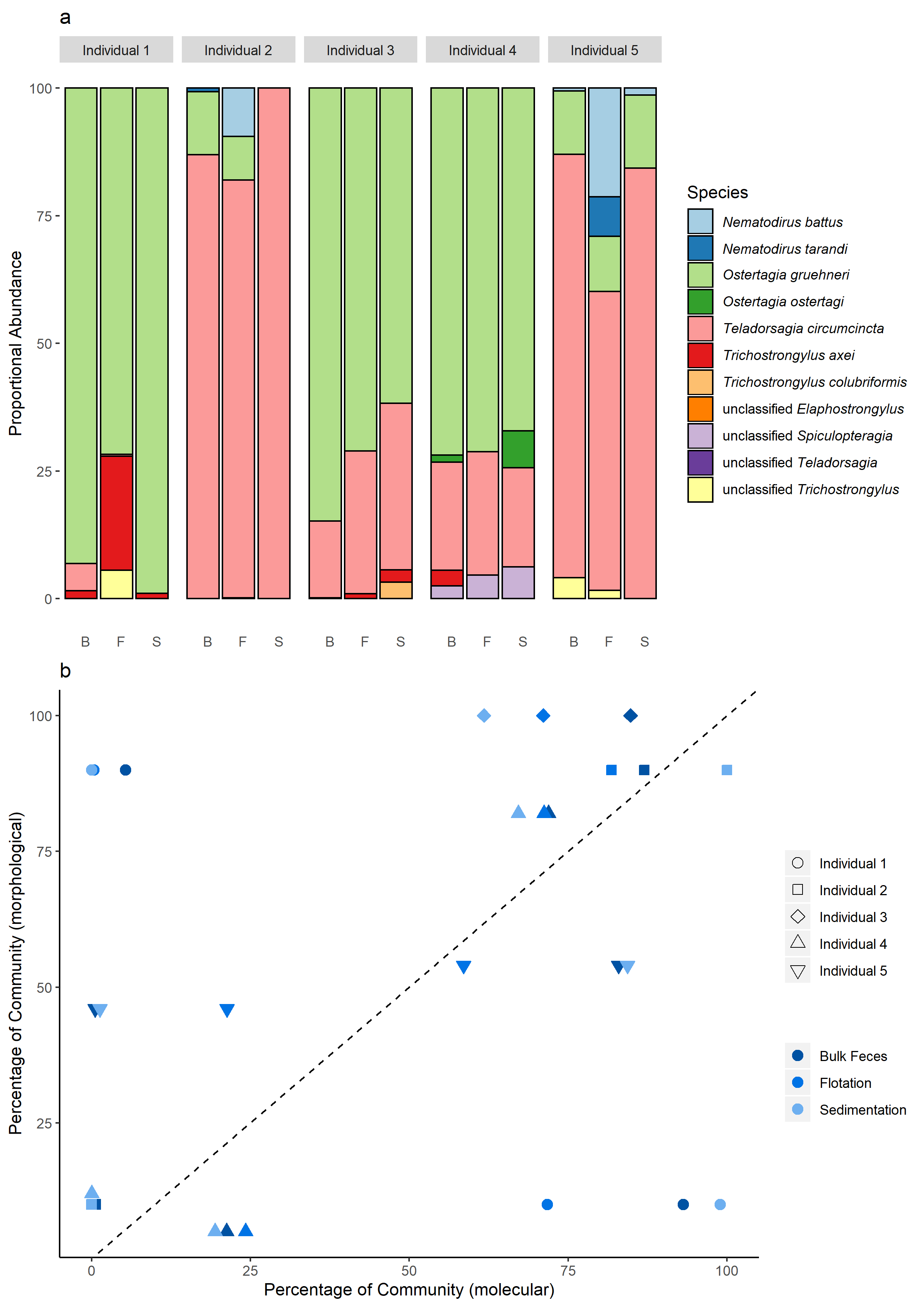

Supplement: Supplementary file 3 — Additional file 3: Figure S2. Proportional abundances of species recovered from faeces samples after flotation and sedimentation treatments (a). Comparison of the proportional abundances of species in GIN communities as determined by morphological identification from abomasum and duodenum samples, and molecular identification from faecal samples (b) B: bulk samples, untreated, F: flotation, S: sedimentation. [file 13071_2021_4935_MOESM3_ESM.png]

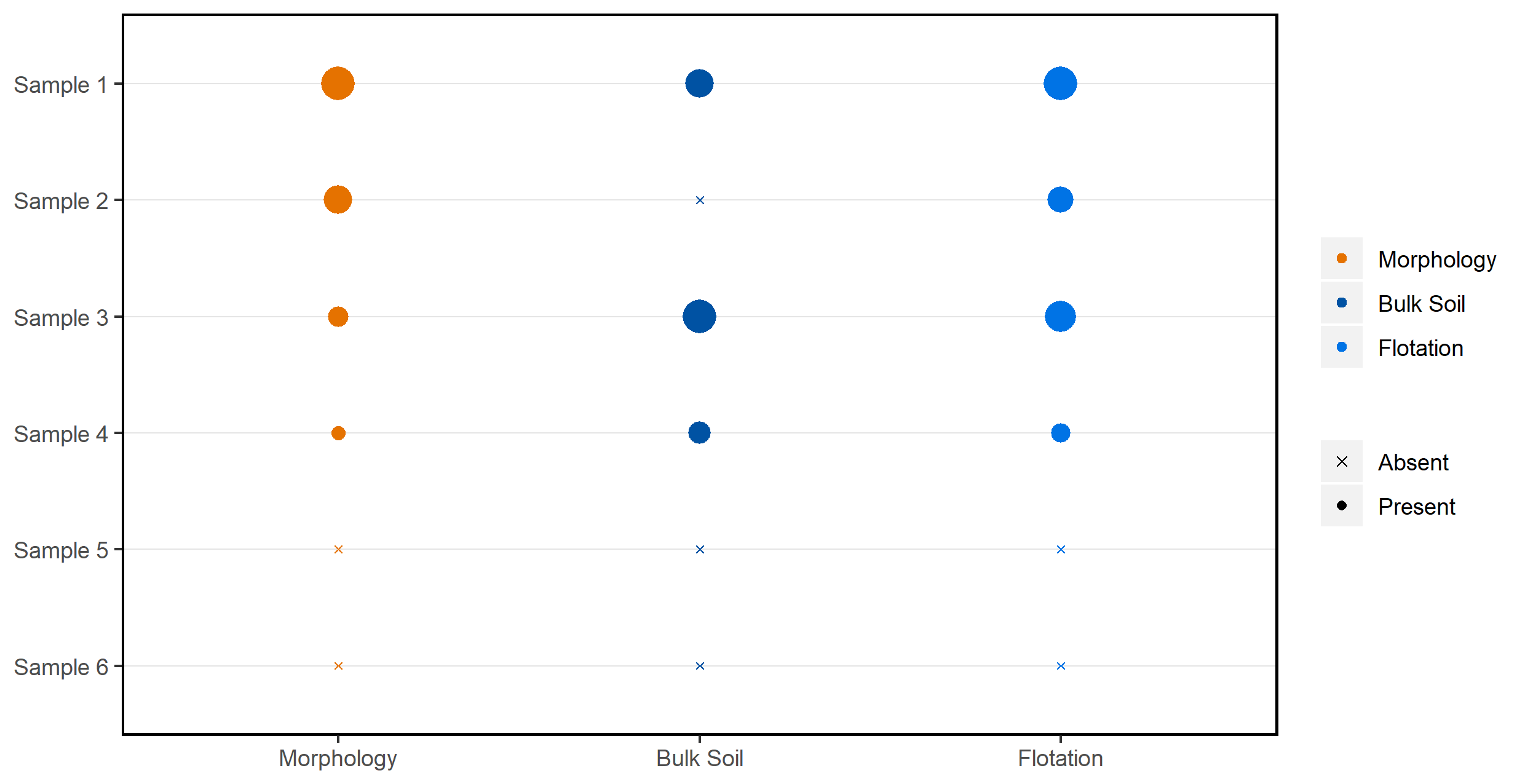

Supplement: Supplementary file 4 — Additional file 4: Figure S3. Detection of Nematodirus battus from soil samples using morphology- and molecular-based approaches. Points are sized relative to the number of N. battus eggs detected (morphology) or the proportional abundance of N. battus reads in the sample (genetic methods). [file 13071_2021_4935_MOESM4_ESM.png]

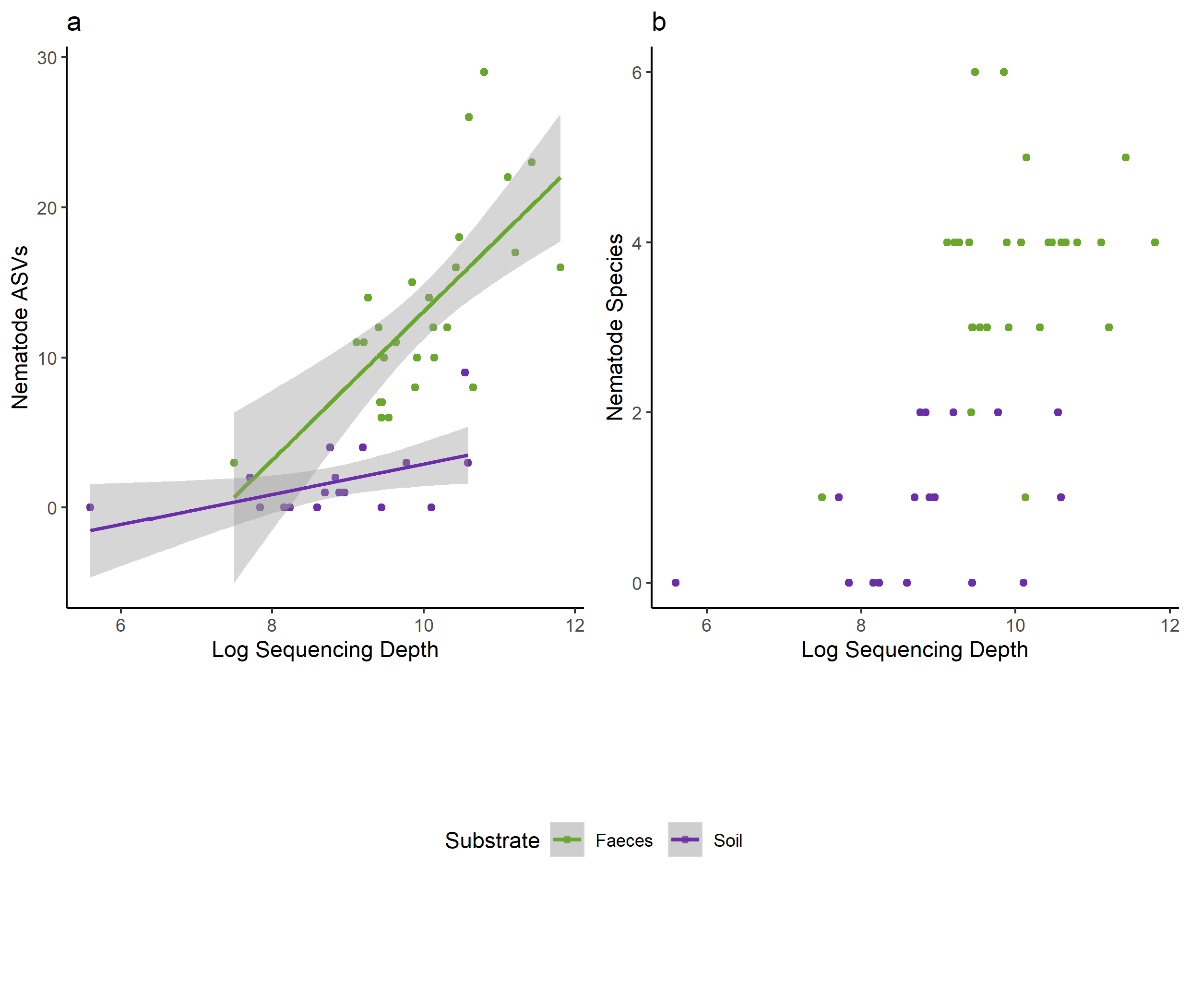

Supplement: Supplementary file 5 — Additional file 5: Figure S4. The relationship between sequencing depth and the number of parasitic nematode ASVs (a) and species (b) recovered from faeces and soil samples. Statistically significant relationships are indicated with fitted lines. [file 13071_2021_4935_MOESM5_ESM.png]
